# Parents’ satisfaction and children's acceptance of nasal compared to oral midazolam for sedation in two consecutive pediatric dental treatments: a randomized controlled study

**DOI:** 10.3389/fdmed.2023.1296823

**Published:** 2023-12-21

**Authors:** Avia Fux-Noy, Qamar Saadi, Aviv Shmueli, Elinor Halperson, Diana Ram, Moti Moskovitz

**Affiliations:** ^1^ Department of Pediatric Dentistry, Faculty of Dental Medicine, Hebrew University of Jerusalem, Israel, Hadassah Medical Center, Jerusalem, Israel; ^2^ Department of Pediatric Dentistry, Hadassah Medical Center, Jerusalem, Israel

**Keywords:** oral midazolam, nasal midazolam, pediatric dental treatment, moderate sedation, premedication acceptance

## Abstract

**Introduction:**

Midazolam, by either the oral or the nasal route, is safe and effective in reducing anxiety and improving behavior in children during dental procedures.

**Aim:**

To compare both children's acceptance and parents’ satisfaction with midazolam premedication, when administered as an oral syrup or as a nasal spray and explore whether there were positive or negative changes in acceptance during consecutive dental treatment visits.

**Methods:**

Randomized controlled study among uncooperative 2–6-year-old children who needed at least two similar dental treatments. On each visit, the acceptability of the medication was assessed as good, fair, or poor. The duration of crying after medication administration was recorded. Additionally, parents were asked to rank their satisfaction with the mode of administration.

**Results:**

The study group included 60 children; 30 patients received midazolam orally and 30 nasally. Route of administration did not correlate with medication acceptance in the first (*p* = 0.11) and second visit (*p* = 0.61). However, in the oral group, medication acceptance of 73% of children deteriorated on the second visit, compared to 33% in the nasal group (*p* = 0.01). Parents of children in the oral group expressed less satisfaction with premedication administered on the second visit than did parents of children in the nasal group, *p* = 0.00. Poor medication acceptance at the first visit (*p* = 0.014) and oral route of administration (*p* = 0.014) were found to be predictors of poor medication acceptance at the second visit.

**Conclusions:**

Acceptance of premedication is expected to deteriorate after the first treatment visit, especially in the oral route of administration. Dentists should consider nasal spray administration for young pediatric patients who need more than one dental treatment. Parents should be prepared for possible resistance or refusal by children.

**Clinical Trial Registration:**

ClinicalTrials.gov, identifier NCT02679781.

## 1. Introduction

Moderate sedation is a frequently used behavior guidance technique for providing comprehensive dental treatment to fearful, uncooperative young children (1). Midazolam is well established as safe and effective in reducing anxiety and improving behavior in children prior to medical and dental procedures (2, 3). Midazolam can be administered by either enteral or parenteral routes. The practice of administering midazolam by either the oral or nasal route is becoming rapidly incorporated into routine pediatric dental care (3–8). A recent systematic review reported no difference between nasal midazolam and other midazolam routes of administration on behavior and sedation level (9). The literature is controversial regarding the advantages and limitations of the different administration routes of midazolam, especially with respect to the ease of administration and patient acceptance (2–12). Although the oral route of administration is prevalent among pediatric dentists, confrontation and frustration often arise when children refuse to accept the sedative medication. Despite efforts to disguise the taste, children can spit or regurgitate the medication when administered orally (10). Contradictions in the literature regarding patient acceptance of nasal midazolam are like those of oral midazolam. On one hand, some authors have reported that the nasal route required less patient cooperation and was a simple, convenient, noninvasive, painless and reliable alternative to oral drug administration (3, 5, 6). On the other hand, other authors reported nasal midazolam to be noxious, painful and poorly tolerated (4, 11–13).

Traditionally, nasal midazolam has been administered as drops with a syringe, which reduces its bioavailability and increases discomfort. When given as a nasal atomized spray, instead of drops, the absorption of midazolam via the nasal mucosa has been reported to be practically complete (83%), because little of the substance is swallowed (14). For the procedural event of drug administration, children who accepted the spray demonstrated a significant reduction in aversive behaviors compared to those children administered with drops (5).

Many studies that assessed the advantages and limitations of using different administration routes for midazolam as premedication were conducted prior to general anesthesia (11, 12, 15). In dental treatment, children receive premedication multiple times. Tolerance and compliance are crucial for success and subsequent acceptance.

Parental attitudes toward various behavior guidance techniques have changed over the last two decades. Parents tend to prefer more positive approaches, and less aversive approaches (16–18). Therefore, parental satisfaction with the premedication route adopted is also important.

In light of this, the study was designed to examine children's acceptance of premedication in two consecutive dental visits, expected changes in acceptance of premedication on the second dental treatment visit, and parental satisfaction with the medicine administration. The primary objective of this study was to compare medication acceptance between patients who received midazolam premedication prior to dental treatment by means of two routes, orally and by nasal spray, and explore whether there were positive or negative changes in acceptance during consecutive dental treatment visit. The secondary objectives were: (1) to compare parental satisfaction with administering midazolam premedication orally as syrup and nasally with a spray. (2) to explore the influence of different variables (such as age, gender, behavior scale, and parental satisfaction) on medication acceptance.

## 2. Materials and methods

### 2.1. Study design

This randomized controlled study was conducted in the Department of Pediatric Dentistry, Hadassah Medical Center, Jerusalem, Israel.

### 2.2. Randomization

Midazolam administration was assigned by a single trained disinterested investigator to one of the groups (oral or nasal) by simple randomization (flipping of a coin) per patient. Randomization was performed after meeting the study inclusion criteria. The children were treated by four residents in pediatric dentistry in their final stage of residency.

### 2.3. Sample size and power calculation

The sample size was calculated for binary primary outcome measures for a non-inferiority trial (non-inferiority limit of 15 percent) using Sealed Envelope (Sealed Envelope Ltd., London, UK) (Sealed Envelope Ltd. 2012. Power calculator for binary outcome non-inferiority trial. Available at: “https://www.sealedenvelope.com/power/binary-noninferior/” Accessed.

November 19, 2019.); 28 teeth per group were required to detect a significant difference for a two-sided type I error at 5% and 90% power. The sample size was increased to compensate for attrition.

### 2.4. Study group

The study inclusion criteria were healthy children (American Society of Anesthesiologists 1 category), aged 2–6 years, uncooperative (Frankl 1-2) in a dental examination (19), with or without previous dental experience, and requiring similar dental treatment in at least two quadrants, with local anesthesia and moderate sedation with midazolam. The dental treatments could include restorations, pulp treatment, stainless-steel crowns, or extractions. The second visit was scheduled 2–4 weeks after the first. Exclusion criteria were enlarged tonsils [Brodsky's grading scale +3 and +4] (20), upper respiratory tract infection and nasal discharge, patients with disabilities or cognitive impairment, and patients who have recently used medication that might interfere with midazolam pharmacokinetics.

### 2.5. Midazolam administration

On the day of treatment, obedience to fasting instructions was verified (no fatty meal for 8 h prior to treatment, no light meal for 6 h prior to treatment and no clear liquids for 2 h prior to treatment, as per the Israeli Division of Dental health guidelines). Ampules of midazolam solution were used (Midolam 5 mg/1 ml, Rafa Laboratories Ltd, Jerusalem, Israel). The oral midazolam dose was 0.5 mg/kg up to a maximum of 10 mg. The medication was mixed with 1 ml of saccharine-sweetened raspberry flavored commercial syrup (Hillel Industry LTD, Israel) to mask its bitter taste and administered using a 5 ml disposable syringe. Nasal midazolam was administered as a nasal spray (MAD Nasal™ Intranasal Mucosal Atomization Device), at a dose of 0.2 mg/kg up to a maximum of 5 mg, up to 1 ml in one nostril.

Acceptability of taking the medication was assessed and recorded by the treating dentist who administered the medication, as described by Baldwa et al. (13) as follows: (1) Good- easily allowing administration; (2) Fair- allowing administration with persuasion; (3) Poor- not allowing administration or administration with restraint. When acceptance was poor children had to be restrained by their parents and/or dentist to receive the medication. The duration of loud crying, if any, after receiving the medicament was measured with a stopwatch by a single observer (QS) waiting outside the premedication room, blinded to the route of administration. Then, parents were asked by the blinded observer to rank their satisfaction with the premedication administering as high, moderate, or low immediately after receiving the midazolam.

Each child waited in the premedication room with his/her parent until signs of onset of sedation were obtained. Then the child entered the treating room and was seated in the dental chair. During treatment, 50% nitrous oxide/ 50% oxygen, according to the rapid induction technique (21), was administered via a nasal hood.

The Houpt scale (22) was used to measure children's overall behavior during dental treatment. The rating was completed by the observer (QS) who was blinded to the route of administration.

### 2.6. Statistical analysis

Data were analyzed in SPSS software (version 28.0.; SPSS, Inc., Chicago, Illinois, USA). Descriptive statistics were produced using means, standard deviations (SD), ranges, frequencies, and percentages. Differences according to route of premedication administration were assessed using the Mann-Whitney and t-test for the continuous variables, and the Chi-square tests for the categorical variables. Differences according to visit were assessed using the Wilcoxon test for the continuous variable, and the Chi-squared tests for the categorical variables. The associations between variables of the first visit and acceptance in the second visit were assessed using t-test and Chi-square tests. Finally, a multiple logistic regression model was used to predict poor acceptance at the second visit. The results were considered significant for alpha less than 5%.

### 2.7. Ethical considerations

The study protocol was approved by the Institutional Human Subjects Ethics Committee (0678-15-HMO). All the procedures performed were in accordance with the ethical standards of the institutional and national research committees. The study protocol was also registered, and the full trial protocol can be accessed at clinicaltrials.gov (registration number: NCT02679781, date of registration: 10/02/2016). Detailed information in simple non-technical language was provided in advance and parents/guardians of all the patients included in the study were requested to sign an informed consent form. No compensation was provided for participation.

## 3. Results

**Figure 1:**
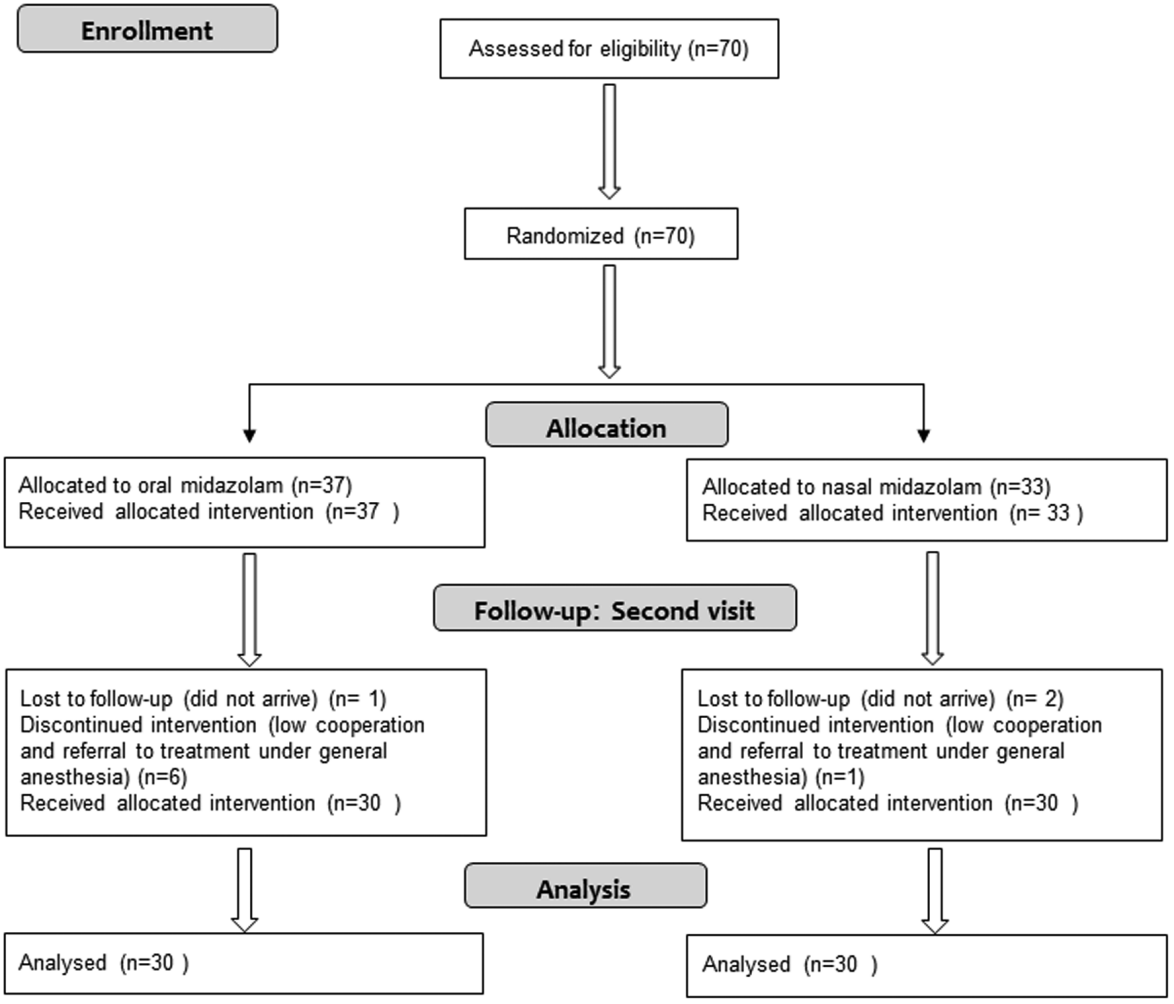
CONSORT flow diagram.

Recruitment was performed during the years 2019–2020. Seventy children were recruited for the study. Of them, 10 were excluded from the analysis: seven due to lack of cooperation and referral to treatment under general anesthesia, six of whom received oral midazolam. Three participants did not attend the second treatment (one of them received oral midazolam). The Consort flow diagram is presented in Figure 1. The study group included 60 children (31 males). The age range was 2–6 years; the mean was 4.36 years (SD = 1.32). The mean weight of the study participants was 16.90 kilograms (SD = 3.94); 30 received midazolam orally and 30 received it intranasally. The two groups matched in age, gender and weight characteristics (Table 1).

**Table 1:** Characteristics of the study participants.

Baseline characteristic		Total study group	Nasal group	Oral group	*p*-value
Age, years	Range	2–6	2–6	2–6	0.529^a^
	Mean (SD)	4.36 (1.32)	4.46 (1.25)	4.25 (1.39)	
Gender	Male *n* (%)	31 (52)	16 (53)	15 (50)	0.796^b^
	Female *n* (%)	29 (48)	14 (47)	15 (50)	
Weight, kg	Mean (SD)	16.90 (3.94)	17.03 (4.03)	16.77 (3.91)	0.796^a^

^a^ *t*-test.

^b^ Pearson Chi-square.

Table 2 presents outcomes according to the route of administration at the first and second treatment visits. Route of administration did not correlate with medication acceptance in the first (*p* = 0.11) and second visit (*p* = 0.61). In the first visit, a higher proportion of patients in the nasal than the oral group cried: 77% vs. 30% (*p* = 0.00). The duration of crying was longer in the nasal group (mean = 0.97, SD = 0.72) than in the oral group (mean = 0.53, SD = 0.90), *p* = 0.01.

**Table 2:** Comparing outcomes according to the route of administration at first and second dental treatment.

			Nasal group		Oral group		*p*-value
			*n*	%	*n*	%	
First dental treatment	Acceptance of premedication	Good	4	13	11	37	0.11^a^
		Fair	18	60	14	47	
		Poor	8	27	5	17	
	Crying (yes)		23	77	9	30	0.00^a^
	Parental satisfaction	High	25	83	27	90	0.70^b^
		Medium	5	17	3	10	
		Low	0	0	0	0	
			Mean	SD	Mean	SD	
	Crying duration (min)		0.97	0.72	0.53	0.90	0.01^c^
	Child cooperation (Houpt)		4.80	0.80	5.17	0.79	0.08^c^
Second dental treatment	Acceptance of premedication	Good	3	10	1	3	0.61^d^
		Fair	12	40	11	37	
		Poor	15	50	18	60	
	Crying (yes)		22	73	21	70	0.77^a^
	Parental satisfaction	High	24	80	8	27	0.00^d^
		Medium	6	20	20	67	
		Low	0	0	2	7	
			Mean	SD	Mean	SD	
	Crying duration (min)		1.37	1.13	2.07	1.82	0.17^c^
	Child cooperation (Houpt)		4.87	0.78	4.93	0.83	0.74^c^

^a^ Pearson chi-square.

^b^ Fisher exact test.

^c^ Mann–Whitney.

^d^ Fisher–Freeman–Halton exact test.

In the second visit, premedication acceptance was poor in 50% and 60% of the participants in nasal and oral groups, respectively, and most children in both groups cried while receiving the premedication. Parents of children in the oral group expressed less satisfaction with premedication administering than did parents of children in the nasal group, *p* = 0.00.

Based on the medication acceptance in each visit, a profile was determined for each patient. Also, a crying profile was determined for each patient (Table 3). In the oral group, medication acceptance of 73% of children deteriorated on the second visit, compared to 33% in the nasal group (*p* = 0.01). The difference was statistically significant also for the crying profile (*p* = 0.00), with more children in the oral group deteriorating and crying on the second visit, none of them improved.

**Table 3:** Acceptance and crying profiles according to the route of administration .

		Nasal group		Oral group		*p*-value
		*n*	%	*n*	%	
Acceptance profile	Good/fair in both visits	9	30	3	10	0.01^a^
	Deteriorated on second visit	10	33	22	73	
	Poor in both visits	8	27	4	13	
	Improved on second visit	3	10	1	3	
Cry profile	No crying in both visits	3	10	9	30	0.00^a^
	Crying at second visit, not at first	4	13	12	40	
	Crying in both visits	18	60	9	30	
	Crying only at first visit, not at second	5	17	0	0	

^a^ Fisher–Freeman–Halton exact test.

Table 4 presents outcomes according to visits within each group. For the nasal group, crying duration was significantly longer at the second visit (*p* = 0.03). For the oral group, significant differences between visits were found in crying frequency (*p* = 0.00), crying duration (*p* = 0.00), and medication acceptance (*p* = 0.00).

**Table 4:** Comparing outcomes between visits within each group.

Nasal group					*p*-value
Crying duration (min) Mean (SD)		1st visit 0.97 (0.72)	2nd visit 1.37 (1.13)		0.03^a^
Crying		2nd visit			0.50^b^
		Yes	NO		
1st visit *n* (%)	Yes	18 (60)	5 (17)		
	No	4 (10)	3 (10)		
Acceptance		2nd visit			0.059^b^
		Good	Fair	Poor	
1st visit *n* (%)	Good	1 (3)	3 (10)	0 (0)	
	Fair	2 (7)	8 (27)	8 (27)	
	Poor	0 (0)	1 (3)	7 (23)	
Parental satisfaction		2nd visit			1.00^b^
		Low	Medium	High	
1st visit *n* (%)	Medium	0 (0)	5 (17)	0 (0)	
	High	0 (0)	1 (3)	24 (80)	
Oral group					*p*-value
Crying duration (min) Mean (SD)		1st visit 0.53 (0.90)	2nd visit 2.07 (1.82)		0.00^a^
Crying		2nd visit			0.00^b^
		Yes	No		
1st visit *n* (%)	Yes	9 (30)1	0 (0)		
	No	12 (40)	9 (30)		
Acceptance		2nd visit			0.00^b^
		Good	Fair	Poor	
1st visit *n* (%)	Good	1 (3)	8 (27)	2 (7)	
	Fair	0 (0)	2 (7)	12 (40)	
	Poor	0 (0)	1 (3)	4 (13)	
Parental satisfaction		2nd visit			
		Low	Medium	High	
1st visit *n* (%)	Medium	1 (3)	2 (7)	0 (0)	
	High	1 (3)	18 (60)	8 (27)	

^a^ Wilcoxon.

^b^ McNemar chi-square.

The effects of the various variables in the first dental treatment on poor acceptance of premedication in the second treatment were assessed (Table 5). A significant association was found between poor premedication acceptance in the second treatment and premedication acceptance in the first treatment (*p*-value = 0.00), crying after premedication administration in the first treatment (*p*-value = 0.022), and parental satisfaction at the first visit (*p*-value = 0.006).

**Table 5:** Effects of the variables of the first dental treatment on the acceptance of premedication in the second treatment .

Variables in the first dental treatment	*N* (%)	Medication acceptance second dental treatment		*p*-value
		Good + Fair *n* (%)	Poor *n* (%)	
Gender:				
Male	31 (100)	12 (39)	19 (61)	0.311^a^
Female	29 (100)	15 (52)	14 (48)	
Route:				0.436^a^
Nasal	30 (50)	15 (50)	15 (50)	
Oral	30 (50)	12 (40)	18 (60)	
Medication acceptance:				0.000^a^
Good	15 (25)	13 (87)	2 (13)	
Fair	32 (53)	12 (38)	20 (63)	
Poor	13 (22)	2 (15)	11 (85)	
Crying after premedication:				0.022^a^
No	32 (53)	10 (31)	22 (69)	
Yes	28 (47)	17 (61)	11 (39)	
Parental satisfaction:				0.006^b^
Medium	8 (100)	0 (0)	8 (100)	
High	52 (100)	27 (52)	25 (48)	
Child behavior (Houpt):				0.793^c^
Fair	2 (100)	1 (50)	1 (50)	
Good	14 (100)	8 (57)	6 (43)	
Very good	27 (100)	11 (41)	16 (59)	
Excellent	17 (100)	7 (41)	10 (59)	
Age (years) [mean ± SD]		4.42 ± 1.26	4.30 ± 1.38	0.726^d^

^a^ Pearson chi-square.

^b^ Fisher exact test.

^c^ Fisher–Freeman–Halton exact test.

^d^ *t*-test.

The multiple logistic regression model found that poor medication acceptance at the first visit (*p* = 0.014) and route of administration (*p* = 0.014) were predictors of poor medication acceptance at the second visit (Table 6). The probabilities of poor premedication acceptance in the second treatment were 9 times greater for oral route compared to nasal, and 30 times greater for poor acceptance at first visit compared to good.

**Table 6:** Multiple logistic regression model for predicting poor medication acceptance at 2nd visit.

	Sig.	Adjusted OR	95% C.I. for OR	
			Lower	Upper
Any crying 1st visit	0.149	3.609	0.631	20.627
Acceptance 1st visit: Good	0.014			
Acceptance 1st visit: Fair	0.008	13.619	1.966	94.336
Acceptance 1st visit: Poor	0.007	29.925	2.559	349.878
Parents’ satisfaction, 1st visit	0.999	673,956,573.099	0.000	
Study group	0.014	9.373	1.568	56.045
Constant	0.001	0.021		

## 4. Discussion

This study compared children's acceptance of oral vs. nasal midazolam for sedation in dental treatment in two consecutive visits and found that although crying was longer and more frequent among children in the nasal group on the first visit, acceptance, crying and parental satisfaction with the oral group was significantly deteriorated on the second visit.

Seventy-seven percent of the children in the nasal group cried after the premedication administration in the first dental treatment and the duration of crying was longer than in the oral group. Similarly, Kogan et al. (11) reported that 77% of children administered nasal midazolam cried after drug administration and concluded that the nasal route causes significant nasal irritation. Other studies also reported that children accepted the drug better when administered orally than nasally (12, 15). In contrast, Musani and Chandan (23) reported that the degree of acceptability of midazolam by both routes was good. Ghajari et al. (6) also reported little or no difference in drug acceptance rates, albeit the drug was administered by force for all their participants.

For both the oral and nasal groups of the current study, acceptance of midazolam was lower in the second visit. Three variables related to the first visit predicted an increased likelihood of refusal to take the medication on the second visit and thus receiving it forcibly. These variables were: lower acceptance of midazolam administration, crying after drug administration, and parental satisfaction. When treating children who express resistance in the first visit, dentists should be prepared for difficulty in administering premedication in subsequent visits and should also prepare the parents for confrontation and frustration. Our results showed that parents were less satisfied with administration of the premedication when it was forced. This emphasizes the importance of coordination of expectations and parents should be prepared for possible resistance or refusal by children, and the possibility that their child will need to be restrained in order to administer the drug. When parents refuse forced administration, other treatment options should be considered. Parental satisfaction was lower in the oral than in the nasal group in the second session. The reason may be that more children in the former received the drug while being restrained by their parents. Providing oral syrup to a resistant child is more time-consuming and more challenging than giving a nasal spray. Kogan et al. (11) reported similar parental satisfaction with four routes of midazolam (nasal, oral, rectal and sublingual). However, they examined overall satisfaction with the sedation rather than with the drug administration, as in the present study.

Limitations of the study: Ten children were excluded from the study, seven of them due to lack of cooperation in the first treatment. They were referred to treatment under general anesthesia. Six of the latter received the midazolam in oral syrup; since most of the dropouts are from the oral group this could have affected the result. Since this study included only two treatment visits, the results may not be applicable to the acceptance of drug administration following three or more visits. The acceptance on the third visit could improve or further deteriorate to not allowing medication administration. In the latter, other pharmacologic methods such as general anesthesia or deep sedation should be considered. In addition, medication administration was delivered by four dentists. Full standardization was not possible and the behavior guidance style of the dentists may cause different acceptance in the child. Also, previous dental experience was not included in the design and could affect the results. Future studies should explore more than two visits and the influence of dental history.

In conclusion, on first dental visits, acceptance of midazolam was similar among pediatric dental patients who received it orally or by nasal spray, however, the nasal route caused more crying. Acceptance of premedication is expected to deteriorate after the first visit, especially when given orally. Parents’ satisfaction with drug administration decreased following the oral route, as children's acceptance decreased. Dentists should consider nasal spray administration for young pediatric patients who need more than one dental treatment since the nasal route requires less patient cooperation and administration is simple and convenient.

## Data Availability

The original contributions presented in the study are included in the article/Supplementary Material, further inquiries can be directed to the corresponding author.
